# Amelanotic subungual melanoma becoming clinically apparent within 24 h after minor blunt trauma: a case report

**DOI:** 10.3389/fmed.2026.1991898

**Published:** 2026-10-09

**Authors:** Huiru Wang, Peng Wang, Yanyan Suo, Kang Lu, Qingfeng Xue, Huahua Li, Rui Gao, Yunchang Guo, Songjia Qu, Xiaowei Cai

**Affiliations:** 1 Department of Dermatology, The 985th Hospital of the Joint Logistic Support Force of the Chinese People's Liberation Army, Taiyuan, Shanxi, China; 2 Department of Pathology, The 985th Hospital of the Joint Logistic Support Force of the Chinese People's Liberation Army, Taiyuan, Shanxi, China; 3 Department of Rehabilitation, The 985th Hospital of the Joint Logistic Support Force of the Chinese People's Liberation Army, Taiyuan, Shanxi, China; 4 Department of Emergency, The 985th Hospital of the Joint Logistic Support Force of the Chinese People's Liberation Army, Taiyuan, Shanxi, China

**Keywords:** acute trauma, amelanotic melanoma, immunohistochemistry, misdiagnosis, subungual melanoma

## Abstract

Amelanotic subungual melanoma (ASM) is a rare variant of nail unit melanoma (NUM) lacking melanin pigmentation, frequently misdiagnosed as a benign post-traumatic or infectious lesion. To our knowledge, ASM becoming clinically apparent within 24 h following minor blunt trauma has not been reported previously. We report the case of a 66-year-old male who presented with a persistent flesh-colored nodule beneath his right thumbnail that was manifested within 24 h after minor blunt trauma caused by nail clippers. The lesion was non-tender, non-pigmented, and unresponsive to 2 weeks of antibiotic therapy. Histopathology revealed diffuse subepidermal proliferation of atypical cells with signet-ring morphology. Immunohistochemistry (IHC) demonstrated positivity for vimentin, HMB45, and Melan-A, and negativity for cytokeratin 5 (CK5) and S-100. Ki-67 was approximately 30%; Breslow thickness was 2.5 mm. Final diagnosis: ASM, AJCC stage IIA (T3aN0M0). Diagnostic excisional biopsy with a 2-mm margin was performed; sentinel lymph node biopsy of two axillary lymph nodes was negative. At 1-year follow-up, no local recurrence or distant metastasis was observed. This case demonstrates that ASM can become clinically evident within 24 h after acute trauma, likely through unmasking of a pre-existing subclinical tumor. For post-traumatic nail lesions unresponsive to antibiotics, early biopsy with a comprehensive IHC panel (including Melan-A, HMB45, and S-100) is essential.

## 1 Introduction

Acral melanoma is the most common histological subtype of cutaneous melanoma among Asian, Hispanic, and African populations (1). Nail unit melanoma (NUM) originates from melanocytes in the nail matrix, nail bed, or periungual epithelium (2). Amelanotic subungual melanoma (ASM), a rare variant lacking typical melanin pigmentation, accounts for approximately 25% of NUM cases (2). Its clinical manifestations—flesh-colored, red, or pink papules—closely resemble benign lesions, representing a significant diagnostic pitfall.

In contrast to cutaneous melanoma, ultraviolet radiation is not a major etiological factor for acral melanoma (3). Recent studies have reported a notable association between trauma and acral melanoma (4, 5), though the precise etiological mechanisms remain to be fully elucidated (6). A large retrospective study of 313 patients found that 26.8% had a documented history of acute trauma before onset, with nail lesions showing the strongest association (7). Furthermore, Teng et al. (8) demonstrated in a multicenter analysis of 468 patients that a history of trauma was an independent risk factor for reduced disease-free survival and overall survival in patients with stage I–II acral melanoma.

Post-traumatic presentations of subungual amelanotic melanoma have been reported previously (9). However, clinical recognition within 24 h of trauma — an exceptionally short interval — has not, to our knowledge, been previously documented. Our literature search of PubMed and Web of Science using combinations of the terms “subungual melanoma,” “amelanotic melanoma,” “trauma,” “acute,” and “nail unit melanoma” through June 2026 did not identify any case with a comparably short trauma-to-recognition interval. This case report provides three clinically relevant insights. First, ASM can become clinically evident within 24 h after minor blunt trauma, a temporal pattern not previously documented. Second, the S-100-negative, Melan-A/HMB45-positive immunophenotype highlights the need for multi-marker IHC panels. Third, this case indicates that, in carefully selected patients who decline or are unsuitable for radical resection, conservative management under close surveillance may offer a reasonable shared decision-making option. Nevertheless, given the limited evidence from a single case, this approach requires further longitudinal follow-up and a larger cohort before its oncological safety can be fully established.

## 2 Case report

A 66-year-old male presented to our dermatology department with a persistent nodule beneath his right thumbnail. The lesion was first noticed by the patient within 24 h after accidental minor blunt trauma to the nail bed while using nail clippers (Day 1). It was a flesh-colored, non-tender nodule without pigmentation or periungual skin changes. After the onset of the lesion, the patient visited several local hospitals and was diagnosed with bacterial soft tissue infection at each visit. He received 2 weeks of systemic broad-spectrum antibiotic therapy and local wound care (Days 1–14). After antibiotic treatment, the superficial purulent discharge over the nodule resolved, but the nodule itself persisted without regression (Figures 1a,b). A potassium hydroxide (KOH) direct microscopic examination for fungal elements was performed at an outside hospital prior to referral and was negative. Fungal culture was not performed.

**Figure 1:**
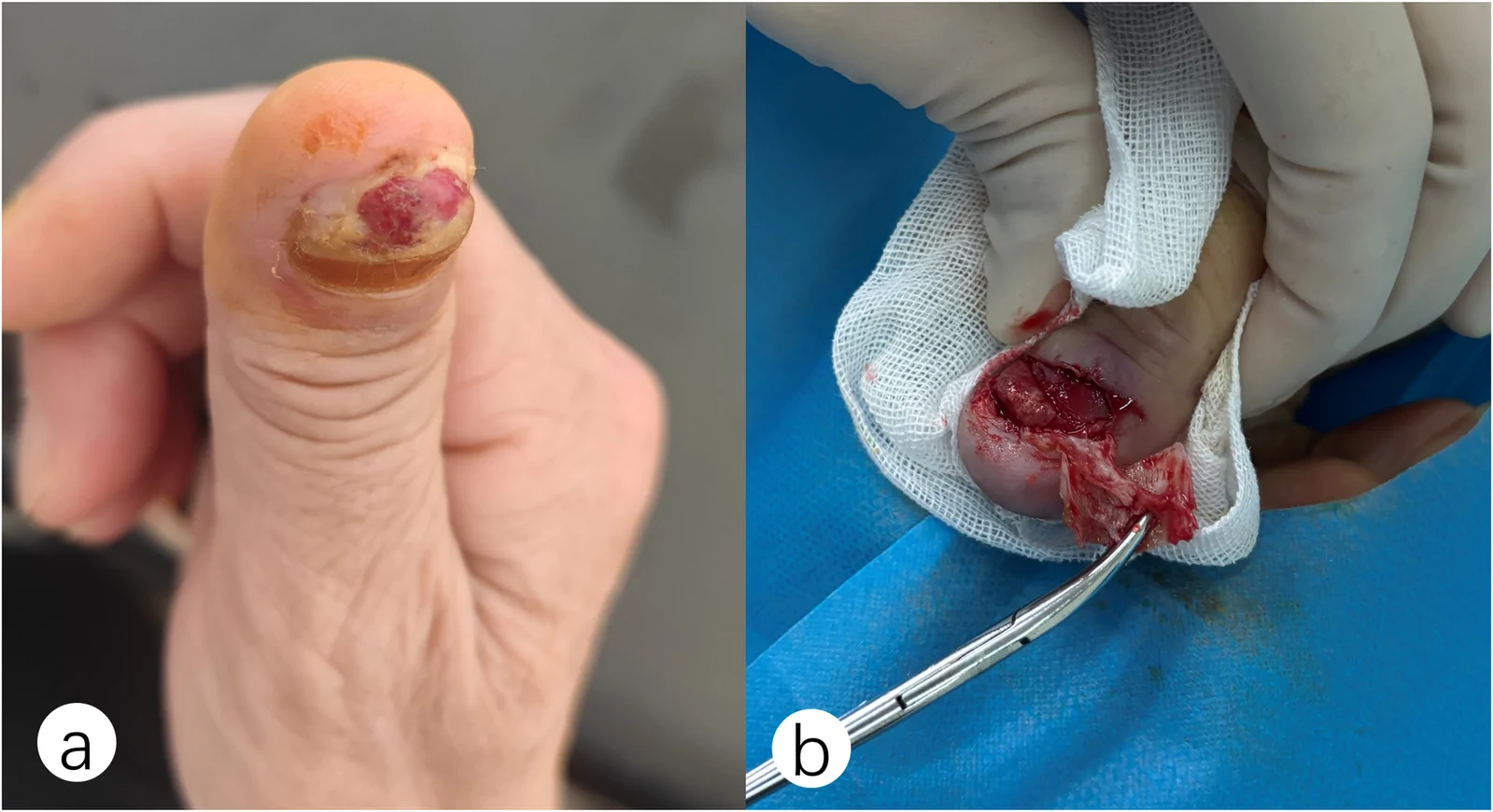
Clinical presentation and intraoperative view. **(a)** A well-defined flesh-colored nodule under the right thumbnail at initial presentation, without pigmentation. **(b)** Intraoperative view showing the flesh-colored nodule on the nail bed after the nail plate was removed.

On physical examination at our department, a solitary, well-circumscribed nodule was observed beneath the right thumbnail (Figure 1a). No longitudinal melanonychia, abnormal pigmentation, or Hutchinson sign was detected. No regional lymphadenopathy was palpated in the right axilla or epitrochlear region. The patient had no significant past medical history, family history of melanoma, or hereditary genetic predispositions.

We performed a diagnostic excisional biopsy under local anesthesia with a 2-mm peripheral margin (Day 15). The nail plate was removed to fully expose the nail bed lesion (Figure 1b). This narrow margin was intentionally limited to diagnostic purposes; for definitive treatment of T3a melanoma, guideline-recommended wide local excision with 1–2 cm margins would be required.

Histopathological examination (Day 22) revealed an epidermal defect and diffuse subepidermal proliferation of atypical cells. The neoplastic cells exhibited signet-ring morphology, eccentrically located nuclei, and prominent nucleoli, with no significant melanin granules observed (Figure 2a, HE,  × 40; Figure 2b, HE, ×200; Figure 2c, HE, ×400). Immunohistochemical (IHC) staining demonstrated positive expression of vimentin, HMB45, and Melan-A, while negative staining was observed for cytokeratin 5 (CK5) and S-100 (Figures 3a,b,c,d,e, ×200). The Ki-67 proliferation index was approximately 30% (Figure 3f, ×200). Breslow thickness was 2.5 mm. No ulceration was identified. Both surgical margins were tumor-free. No vascular or perineural invasion was identified.

**Figure 2:**
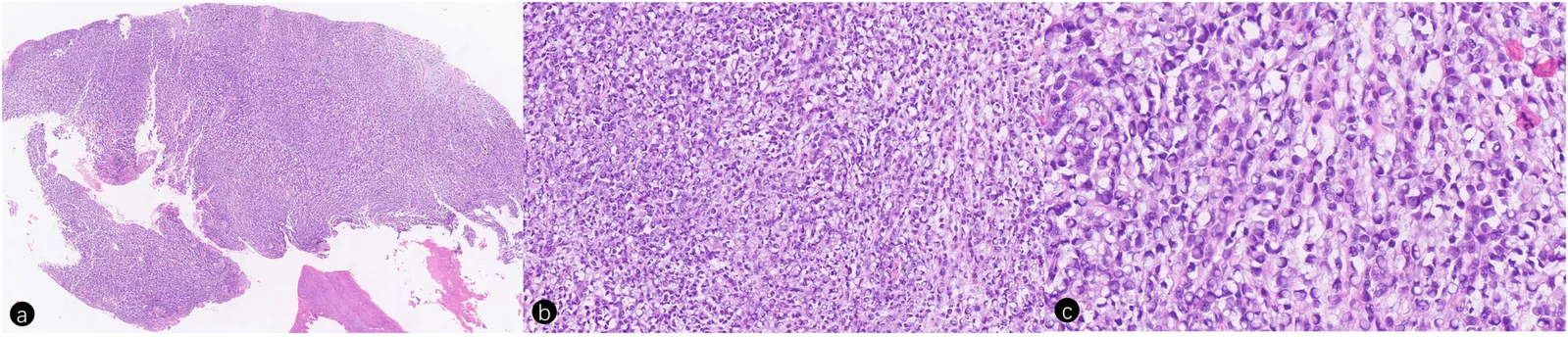
Histopathological examination. **(a)** Epidermal defect and diffuse subepidermal proliferation of atypical cells (HE, ×40). **(b)** Neoplastic cells exhibiting signet-ring morphology, eccentrically located nuclei, and prominent nucleoli, without significant melanin granules (HE, ×200). **(c)** High-power view of atypical cells (HE, ×400).

**Figure 3:**
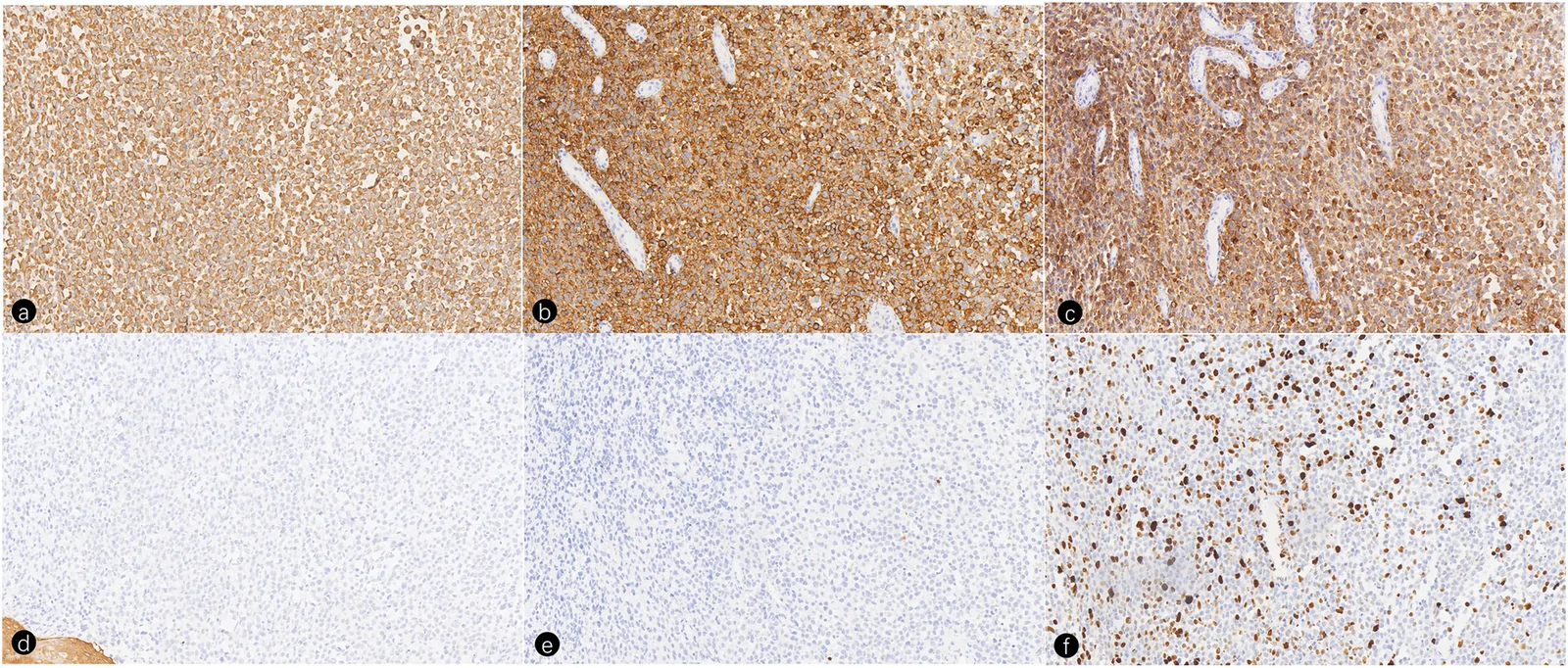
Immunohistochemical staining (×200). **(a)** Vimentin (+). **(b)** HMB45 (+). **(c)** Melan-A (+). **(d)** Cytokeratin 5 (CK5) (-). **(e)** S-100 (-). **(f)** Ki-67 proliferation index approximately 30%.

## 3 Diagnostic assessment, therapeutic intervention, follow-up, and outcomes

After histopathologic confirmation, comprehensive staging was performed (approximately Day 32), including whole-body superficial lymph node ultrasonography and sentinel lymph node biopsy (SLNB). SLNB was performed using indocyanine green (ICG) near-infrared fluorescence tracing. Two sentinel lymph nodes from the right axillary region were identified, harvested, and examined; both were negative for metastasis. No regional or distant metastasis was identified. Per the AJCC 8th edition criteria, the patient was staged as IIA (T3aN0M0).

Wide local excision with 1–2 cm margins, as recommended by current guidelines for T3a melanoma, was discussed with the patient. After thorough counseling regarding the risks of potential local recurrence and distant metastasis associated with conservative management, the patient declined further surgery. His primary reason was that he felt the tumor nodule had been completely removed and preferred close surveillance over additional intervention. Adjuvant systemic therapy was also discussed; the patient similarly declined. He was informed of the elevated recurrence risk associated with declining both definitive surgery and adjuvant therapy.

At the last follow-up (1 year postoperatively), the patient remained stable with no signs of disease progression. Regular surveillance with clinical examination and ultrasonography continues.

## 4 Discussion

### 4.1 Diagnostic challenges of post-traumatic ASM

Due to the lack of typical melanin, the diagnosis of ASM is a challenge in both dermatology and oncology (10). This lesion is frequently misdiagnosed as post-traumatic infection, foreign body reaction, pyogenic granuloma, or subungual hematoma in daily clinical practice (2). In our case, the patient was misdiagnosed with bacterial infection and received unnecessary antibiotic treatment for 2 weeks, and fungal infection was considered and excluded by KOH examination. This fully reflects the common diagnostic pitfall of post-traumatic ASM (11, 12, 25). Misdiagnosis and delayed diagnosis directly lead to tumor progression; delayed diagnosis is the most important risk factor for poor prognosis in patients with acral amelanotic melanoma (10, 11). Most patients are diagnosed at an advanced stage with thicker Breslow thickness, higher ulceration rates, and a greater risk of lymph node metastasis, resulting in significantly lower 5-year overall survival compared to patients with pigmented melanoma (12, 13).

Furthermore, the combination of signet-ring cell morphology and S-100 negativity in our case added considerable diagnostic complexity. Signet-ring cells can be seen in a variety of malignancies, including metastatic adenocarcinoma (particularly from the gastrointestinal tract or breast), epithelioid sarcoma, and certain lymphomas. The IHC panel was critical for differential diagnosis: positivity for vimentin, HMB45, and Melan-A, together with negativity for CK5 (a cytokeratin marker that helps exclude carcinoma), supported the diagnosis of melanoma despite S-100 negativity. The absence of glandular differentiation and negative cytokeratin expression helped differentiate this lesion from metastatic adenocarcinoma. The diagnosis of melanoma was supported by the combined histopathological and immunohistochemical findings, including positivity for melanocyte-specific markers.

### 4.2 Trauma as a revealing rather than carcinogenic factor

The relationship between trauma and melanoma has been controversial, but recent studies have reported and further clarified their correlation (5, 14). Lee et al. (7) found that 26.8% of acral melanoma patients had documented acute trauma before onset, with nail lesions showing the closest association (42.0% for fingernail, 36.1% for toenail melanoma). Conway et al. (2) reported that 21.8% of NUM patients had previous trauma history.

Our case is extremely rare because a malignant mass clinically manifested within only 24 h of acute trauma. Melanocyte transformation requires a long-term, multistep molecular process involving gene mutation, dysregulated proliferation, invasion, and angiogenesis (15, 16). The patient did not have sufficient time for these changes. Some melanomas may arise from melanocytes that already harbor multiple genetic alterations *in situ* but remain clinically invisible (17). We hypothesize that clinically undetectable subungual melanoma *in situ* was already present before trauma. Acute blunt trauma induced local edema, inflammation, and increased vascular permeability, thereby unmasking the tumor. Importantly, we propose that trauma served as a ‘revealing factor’ rather than a ‘carcinogenic factor’ — a hypothesis that warrants further investigation.

It should be noted that the Breslow thickness of 2.5 mm (AJCC T3a) classifies this tumor as intermediate-thickness rather than early-stage. The acute temporal profile (24 h from trauma to visibility) refers to the rapidity of clinical manifestation, not pathological staging.

### 4.3 Pivotal role of immunohistochemistry

S-100 is traditionally a highly sensitive marker for melanoma. However, it is not universally positive in nail unit and amelanotic subtypes. Our case was S-100-negative yet Melan-A/HMB45-positive. The Mayo Clinic review (3) noted signet-ring cell melanoma can be S-100-negative, matching our case. Conway et al. (2, 18) demonstrated that Melan-A/MART-1 and HMB45 exhibit higher diagnostic sensitivity (>95%) than S-100 (83.1%) in NUM. Misiag et al. (19)further showed that some amelanotic melanomas show S-100 loss while retaining Melan-A and HMB45 expression, related to dysregulation of the melanin synthesis pathway. In amelanotic melanoma, expression of melanin synthesis-related genes such as MITF and tyrosinase are downregulated, leading to blockade of melanin production, but tumor cells still retain melanocyte lineage differentiation markers (19). A recent case report by Shi et al. (20) described an HMB-45-negative subungual amelanotic melanoma, further underscoring the variability of melanocytic marker expression in this entity and reinforcing the need for multi-marker panels.

The Ki-67 proliferation index of approximately 30% in our case warrants particular attention. In cutaneous melanoma, a Ki-67 index exceeding 20%–25% has been linked to significantly higher recurrence rates and shorter disease-free survival (21). At 30%, this tumor falls into the highly proliferative category, suggesting substantial metastatic potential. The discordance between this elevated proliferation marker and the favorable 1-year outcome underscores the need for continued long-term surveillance, as late recurrence remains possible in tumors with high proliferative activity. Ki-67 should be interpreted alongside other prognostic factors — including Breslow thickness, ulceration status, and mitotic rate — rather than in isolation.

Therefore, for clinically suspected amelanotic melanoma, a comprehensive IHC panel including Melan-A, HMB45, and S-100 must be used to avoid false negatives. When atypical morphologies such as signet-ring cells are present, additional cytokeratin markers should be included to exclude carcinoma.

### 4.4 Conservative surgical management

Treatment of NUM has evolved toward conservative and function-preserving approaches (2, 22, 23). Historically, radical amputation was standard. However, Jo et al.'s (24) meta-analysis showed no significant difference in local recurrence between conservative excision and amputation for *in situ* or minimally invasive NUM, with superior functional outcomes. It is important to clarify that the 2-mm margin in our case was a diagnostic excisional biopsy, not a definitive therapeutic resection. Current guidelines recommend 1–2 cm margins for T3a melanoma. Nevertheless, our patient met several favorable criteria: clear surgical margins on diagnostic biopsy, no vascular or perineural invasion, and Ki-67 of approximately 30%. We did not perform further radical surgery based on the patient's wishes, and no recurrence was observed during the one-year follow-up period. However, this favorable short-term outcome should be interpreted with caution and does not support the conclusion that conservative excision provides adequate oncological disease control in intermediate-thickness melanoma. Long-term surveillance remains essential.

### 4.5 Limitations

This single case report has inherent limitations in generalizability. First, the 1-year follow-up is insufficient for long-term oncological safety assessment. Second, the 24-hour temporal claim is based on the patient's self-report and relies on his recall of a lesion-free nail bed prior to trauma. No objective pre-trauma clinical examination or photograph is available to independently verify the absence of a pre-existing lesion. This introduces the possibility of recall or attention bias. However, the biologically implausible nature of *de novo* 24-hour tumorigenesis — combined with a Breslow thickness of 2.5 mm — strongly supports our hypothesis that a pre-existing subclinical tumor was unmasked by trauma rather than generated *de novo*. Third, dermoscopic examination was not performed. As dermoscopy is a valuable tool for the evaluation of nail unit pigmented and non-pigmented lesions (25), its absence represents a limitation of the clinical workup in this case. Fourth, the intermediate Breslow thickness limits the applicability of conservative surgery observations. Fifth, the patient declined guideline-recommended wide local excision and adjuvant therapy, and the 1-year disease-free interval should not be interpreted as evidence of oncological equivalence to definitive treatment. Finally, mutation testing for BRAF, NRAS, and KIT was not performed. Knowledge of the mutational status would have provided additional prognostic information and could have informed targeted therapeutic options should disease progression occur. Prospective studies with larger cohorts and extended follow-up are needed.

## 5 Conclusion

This rare case demonstrates that ASM can become clinically evident within 24 h after acute trauma, and is highly susceptible to misdiagnosis as benign post-traumatic infection. For non-healing nail lesions after trauma that are unresponsive to antibiotic treatment, early biopsy and histopathological examination are strongly recommended. For the diagnosis of amelanotic melanoma, a panel of IHC markers including Melan-A, HMB45, and S-100 should be used to avoid missed diagnosis, particularly when atypical features such as signet-ring morphology are present. In this case, following diagnostic excisional biopsy with clear margins and a disease-free status at 1-year follow-up, the patient has opted for close surveillance. However, this approach should not be considered oncologically equivalent to guideline-recommended surgical management, and adherence to standard-of-care treatment should be emphasized whenever possible. Long-term and regular follow-up remains essential.

## Data Availability

The original contributions presented in the study are included in the article/Supplementary Material, further inquiries can be directed to the corresponding author/s.
